# Molecular Dynamics Simulation of Coiled Carbon Nanotube Pull-Out from Matrix

**DOI:** 10.3390/ijms23169254

**Published:** 2022-08-17

**Authors:** Feng Huang, Shuai Zhou

**Affiliations:** 1 College of Materials Science and Engineering, Chongqing University, Chongqing 400045, China; 2 State Key Laboratory of Mountain Bridge and Tunnel Engineering, Chongqing Jiaotong University, Chongqing 400074, China; 3 School of Civil Engineering, Chongqing Jiaotong University, Chongqing 400074, China

**Keywords:** composites, coiled carbon nanotubes, interfacial properties, molecular dynamics simulations

## Abstract

The interaction between coiled carbon nanotubes (CCNT) and the polymer matrix is important in the mechanical, thermal, and electrical properties of the CCNT reinforced nanocomposite. In this study, molecular dynamics (MD) simulations were performed to study the interfacial characteristics of polymer nanocomposites (PNCs). Furthermore, the influence of the geometries of the CCNTs on the load transfer mechanism is evaluated. Pullout simulations considering different geometries of CCNTs are carried out to examine the tensile force and the interfacial shear stress (ISS). The results reveal that the maximal tensile force is reduced by increasing CCNT inner diameters, increasing the helix angles, and decreasing nanotube diameters. The distance between CCNTs and the polymer matrix is varied, and the interfacial distance favors greater ISS. Decreasing the inner diameter of the CCNT, the helix angle, and the tube diameter increases the ISS. The enhancement mechanism of CCNT/polymer composites has also been illustrated. Due to a lack of experimental results, only numerical results are given. The present study helps to understand the interfacial adhesion behavior between the polymer matrix and CCNTs and is expected to contribute to the development of CCNT reinforced polymer composites.

## 1. Introduction

Since their discovery in 1991, carbon nanotubes (CNTs) have attracted a lot of attention in most areas of science and engineering [1]. Compared with the straight CNTs, some other kinds of CNTs with curvature have received less attention. They have diverse morphologies as well as unique physical and chemical properties with the potential applications in engineering [2,3]. One kind of them is coiled carbon nanotubes (CCNTs). The straight and curved CNTs are planar structures, while CCNTs can spiral in three-dimensional space. CCNTs exhibit excellent mechanical and electrical properties because of the combination of coil morphology and properties of CNTs [4].

Some research has focused on the mechanical properties of CCNTs. A CCNT behaves like an elastic spring [5]. The CCNT-contained composite has great mechanical properties. Experimental results prove that CCNT/epoxy composites have great Vickers hardness values, Young’s moduli, and tensile strengths. The performance of the CCNT/epoxy adhesive is outstanding due to the enhancement of the mechanical interlocking effect between CCNTs and epoxy. This effect leads to a greater Vickers hardness value, Young’s modulus and lap joint shear strength when compared with the MWNT/epoxy type [6]. It is found that the elongation of the helical composite fibers can reach 100–300%, depending on the pitch of the helix and the tensile strain rate [7]. Meanwhile, molecular mechanics and molecular dynamics are used to investigate the elasticity, plasticity, vibration, and fracture of CCNTs [8,9,10,11,12,13,14,15]. The mechanical performance of CNT-reinforced composites is significantly influenced by the interfacial properties between the CNT and the matrix [1]. A higher interfacial shear stress (ISS) leads to a better stress transfer from the polymer to the reinforcing CNTs [16]. Different parameters determine the reinforcement effect of the CNTs [17,18]. Several experimental studies have been conducted to investigate the interfacial characteristics of the CNT/polymer interface using both direct methods such as pullout or microdroplet tests, and indirect methods such as fragmentation tests or micro-Raman spectroscopy [19,20,21,22,23]. However, only a little research concentrates on the interfacial properties of the CCNT/polymer interface.

Determining their impact on the interfacial properties is essential in understanding how reinforcing mechanisms work. However, it is challenging to measure it experimentally via direct pullout tests [24]. As a result, different analytical and numerical techniques are considered to solve this problem. Some analytical models for the pullout process of wavy CNTs have been developed. The models are capable of predicting axial as well as interfacial shear stresses along a wavy CNT embedded in a matrix. Based on the pullout modeling technique, the effects of waviness, aspect ratio, CNT diameter, volume fraction, Poisson’s ratio, and matrix modulus on axial and interfacial shear stresses have also been analyzed in detail [25,26]. With either a constant or Coulomb friction interface, the pull-out model predicts higher pullout forces as the fiber curvature increases, with zero fiber curvature (a straight fiber) producing the lowest pullout forces. Fiber curvature effects are more pronounced, however, for the Coulomb friction model than for the constant friction model because it considers radial compressive stresses at the fiber/matrix interface [27]. Model predictions of wavy CNT-contained composites indicate that increases in nanotube curvature raise the peak bridging stress but also decrease the average pullout lengths of wavy CNTs. The overall effect can be a reduction in toughness as nanotube curvature increases, depending on the chosen parameters, including interfacial friction properties, nanotube and matrix modulus, and even crack opening [28]. The continuum theory is often applied in the pullout test. Systemic analyses of the pullout behavior of a helical fiber from an elastic matrix via finite element method simulation have been presented previously, with the implications regarding the underlying toughening mechanism of helicoid microstructures. Through their uniform curvature and torsion, helical fibers can provide a high pullout force and large interface areas, resulting in high energy dissipation that accounts, to a large extent, for the high toughness of biological materials [29]. An analytical fiber pullout model tailored for carbon nanotube reinforced polymer composites has been developed based on some classical models. Results show that the required axial stress to pull out a straight CNT at cryogenic temperatures is more than six times greater than that required at room temperature. Some other parameters, such as the length of a CNT and the modulus of the polymer, also influence the stresses in the CNT/polymer model [30]. The shear-lag model of CNTs is performed in the context of linear elasticity for axisymmetric problems. The numerical results reveal that the nanotube aspect ratio is a critical controlling parameter for nanotube-reinforced composites [31]. However, many assumptions introduced in these analytical studies undermine the accuracy of their findings by neglecting the discrete nature of CNTs and assuming CNTs to be homogenous, linearly elastic, and isotropic. MD is an effective method to deal with the problem without these unnecessary assumptions [32,33,34]. It has been widely applied to investigate the interfacial properties between polymer and nanocarbon materials, such as single-layer graphene [35], double-layered graphene [36], functionalized graphene [37], flattened carbon nanotubes [38], functionalized carbon nanotubes [39], multi-walled carbon nanotubes [40], CNT bundles [41], multi-walled carbon nanotubes [42], and single-layer diamonds [43]. The results prove that MD can simulate the interfacial properties with high precision and analyze the underlying toughening mechanism. A series of pullout simulations of carbon nanotubes has been carried out to investigate the interfacial properties between a CNT and a polymer matrix for two-phase CNT/polymer nanocomposites with only consideration of van der Waals interaction. The effects of nanotube length, diameter, and wall number on the pullout processes are studied, which indicates that the pullout force related to interfacial properties is independent of nanotube length but is proportional to nanotube diameter [33]. The work is further extended using united-atom molecular dynamics [34] and an atomistic-based continuum multiscale modeling technique [44]. Using MD, the interfacial properties have been investigated. The pullout force of the wavy CNTs is significantly higher than its straight counterpart and rises further with the increase in the waviness of the CNTs. This is attributed to the added pullout energy dissipated in straightening the CNTs during the pullout process [45]. The key parameters influencing the ISS are the CNT radius, followed by the temperature and pulling velocity, respectively [46]. However, no molecular dynamics simulation has been carried out to examine the influence of geometric parameters of CCNTs on the interfacial properties of the CCNT/polymer interface by pullout tests to the best of our knowledge. Polyethylene (PE) is one of the most widely used organic materials [47] and is often used in the CNT composite [48,49,50,51]. Here, PE is adopted to represent the polymeric matrix.

This research investigates the application of CCNTs in the polymer composite for reinforcement. In Section 2, the pullout behavior of CCNTs and straight CNTs is simulated. The influence of parameters of CCNTs on the interface. The MD model is developed in Section 3. Finally, the conclusions of the present study are summarized in Section 4.

## 2. Results and Discussion

**Figure 1:**
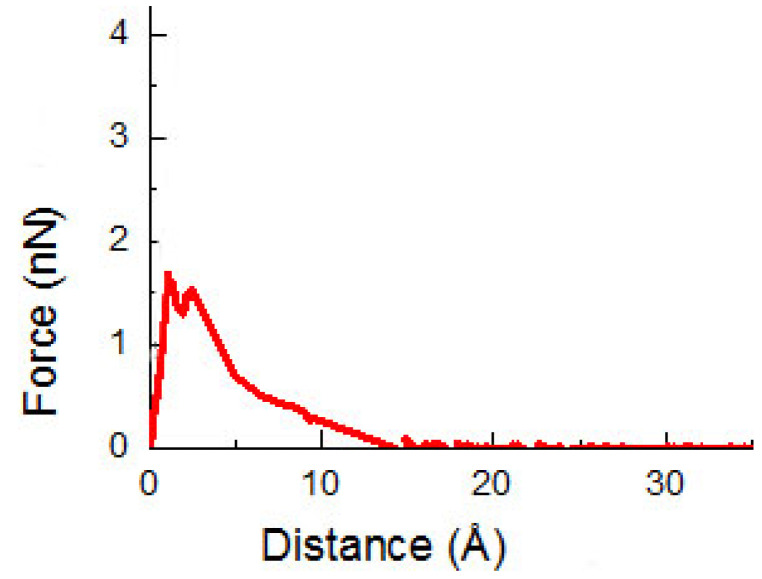
The pullout force versus displacement curve of the CNT with a diameter = 0.81 nm pulled out from the PE matrix.

In the beginning, the CNT(6, 6) with a diameter of 8.14 Å is built and pulled out from the PE matrix. Figure 1 presents the pullout force versus the displacement. The pullout force quickly rises to the maximum force, and then it decreases until it becomes zero when the CNT is pulled out from the PE matrix. The ISS is 132 MPa, which is close to the result ISS = 134 MPa in previous research [33]. Further comparison of the CCNT/PE interface will be conducted in the following research after more experimental results are obtained.

**Figure 2:**
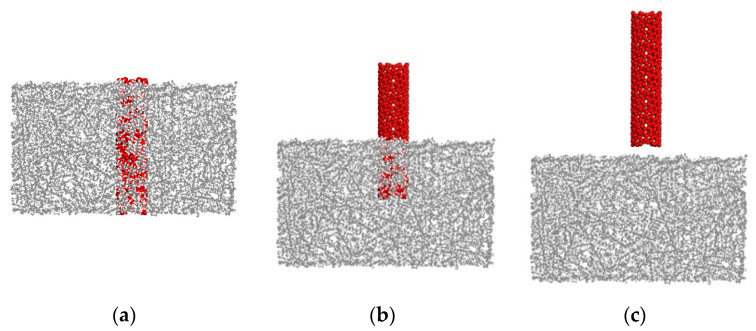
Snapshots of the CNT/PE composite during the pullout process. (**a**) 0 Å (**b**) 25 Å (**c**) 55 Å.

**Figure 3:**
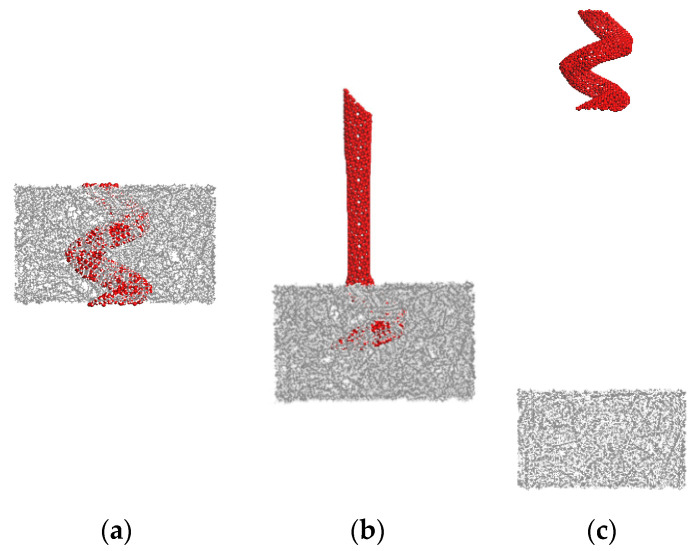
Snapshots of the CCNT/PE composite during the pullout process. (**a**) 0 Å (**b**) 80 Å (**c**) 200 Å.

Two MD simulations of the pullout test of the straight CNT and the CCNT were conducted to determine quantitatively the effect of shape on the pullout force and ISS. The pullout process is completed when the fully embedded nanotube is pulled out from the matrix. Figure 2 shows snapshots of the pullout simulation of a straight CNT. The CCNT, which has the same tube diameter as the straight CNT, is pulled out in Figure 3. The corresponding geometry and numerical results are displayed in Table 1.

From Figure 2 and Figure 3, the total length of the CCNT after being straightened is much longer than that of the straight CNT, even though the heights of RVE in the two cases are the same. The CCNT becomes straight during the pulling process, which consumes more energy considering that its initial configuration is coiled. The deformation of CCNTs contains three stages from Figure 3. Firstly, this pullout stage is characterized by the unfolding and stretching of the CCNT. Then, debonding occurs at the CCNT/matrix interface, beginning to propagate stably along the helical interface. When the crack length reaches a critical value, the crack propagation becomes unstable and usually leads to sudden interface debonding. Finally, frictional sliding of the CCNT begins until it is pulled out. The CCNT gradually restores its initial length and shape. After the CCNT is pulled out from the PE matrix in Figure 3c, it becomes coiled again.

**Figure 4:**
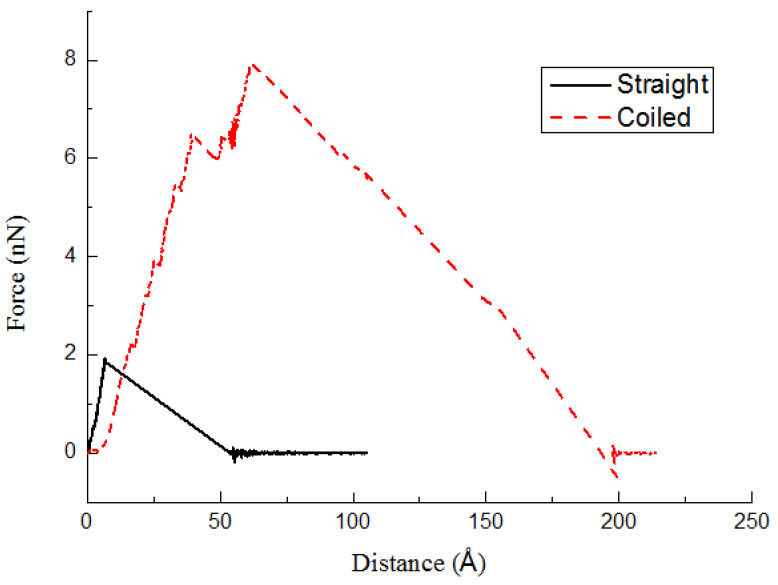
Variation of pullout force throughout the pullout process of CCNTs from the PE matrix.

Figure 4 shows the variation of the pullout force during the simulation. For straight CNTs, the applied pullout force is only responsible for overcoming the non-bonded interactions between the CNT’s atoms and the surrounding polymer molecules. However, for CCNTs, additional work is required to overcome the resistance of the CCNT against shape change and the accompanying change in the potential energy of the deformed CCNT. It can be seen from Figure 4 that a typical force–displacement curve of the pullout process of the CCNT includes three regions, which are similar to those found in Figure 3. During the unfolding and stretching of the CCNT, the elastic section is witnessed and no damage occurs in the beginning. Then, cracks occur at the CCNT/PE interface and quickly propagate after the pullout force increases to a certain value, resulting in a generally fluctuating force–displacement relationship. The crack propagation leads to a sudden interfacial debonding, which causes a decrease in the tensile force. Finally, the tensile force gradually reduces and fluctuates around 0 nN since the frictional sliding of the CCNT continues until the CCNT is pulled out. Previous experimental results showed that CCNT/polymer composites were much stiffer than straight CNT/polymer composites [6]. The interfacial properties may be influenced by many factors. This phenomenon can be well explained by the current model. Since CCNTs are more difficult to pull out, the stress transfer efficiency between CCNT/polymer composites is better than that in straight CNT/polymer composites. CCNTs are able to significantly increase the interfacial shear strength because of strong mechanical interlocking effect. It consequently leads to a greater stiffness in CCNT/polymer composites. Hence, CCNTs are very effective reinforcement for polymers.

The pullout force required to debond CCNTs can be simply described by adding a force *F_coil_* induced by CCNTs onto the force responsible for overcoming the van der Waals interactions as displayed in Equation (1).

*F*_*pull-out*_ = *F*_*vdW*_ + *F*_*coil*_ (1)

**Figure 5:**
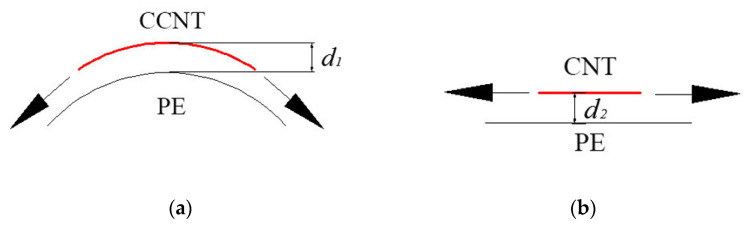
The CCNT and straight CNT on the PE matrix. (**a**) CCNT (**b**) CNT.

There are many factors influencing the *F_coil_*. Firstly, with the curvature, there is a component of force in the CCNT towards the center of the cycle as displayed in Figure 5a. Hence, the distance *d*_1_ in Figure 5a is less than *d*_2_ in Figure 5b. Decreasing the distance between the CCNT and the PE matrix tends to increase the interaction in that area. Hence, the interactions between the CCNT and PE are greater than those between the straight CNT and PE, which influences and contributes to *F_coil_*. Secondly, an extra force is needed to straighten the CCNT during the pullout process, as shown in Figure 3b, which raises the tensile force. Thirdly, in contrast to a straight CNT, each section of the CCNT is subjected to twisting, bending, and axial extension deformations due to the curvature and torsion, leading to a complex stress field. The curvature and torsion of the CCNT induce a combination of the axial shear stress and the circumferential shear stress, which generates a much higher pullout force than that of the straight CNT.

It is clear that the area below the force–displacement curve of the CCNT/PE composite is much greater than that of the CNT/PE composite from Figure 4. The helical shape leads to the greater length of the CCNT after it is straightened. Hence, the actual area between the CCNT and the matrix is much greater than that between the CNT and the matrix. Meanwhile, the tensile force in the CCNT/PE interface is greater than that in the CNT/PE interface from the above analysis. Considering these factors, the helical shape of the CCNT results in greater energy dissipation during the pullout process of the CCNT, even several times greater than that of the CNT reinforced composite. In CNT reinforced composites, the energy dissipation of CNTs during the pullout process is the main contributor to the toughness of the composite. Therefore, the helical shape of a CCNT at the microscale can be considered as one of the main sources of the high toughness of the CCNT reinforced composite.

The obtained pullout force from Equation (1) can be used to calculate the ISS. Table 1 summarizes the results obtained in two different cases. The ISS is also noted to rise in the CCNT/PE interface from Table 1. The ISS of the CNT/PE interface is 127 MPa, which is in good agreement with the value of 128 MPa calculated by Li et al. [33]. The additional interactions arosing from the added CCNTs significantly improve the peak pullout force and produce those observed characteristics in simulations.

### 2.1. Effect of Inner Diameters of the CCNT

**Figure 6:**
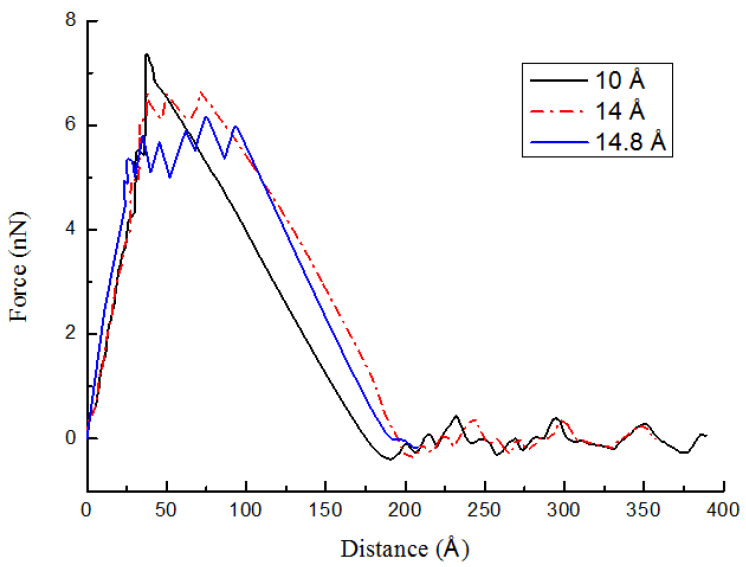
Pullout profiles for CCNTs with different inner diameters.

The inner diameter of a CCNT affects its macroscopic reinforcement properties. Here, three cases are investigated. The geometry of related CCNTs and the summary of the pullout results are listed in Table 2. An initial comparison of the pullout profiles for all three inner diameters of CCNTs is displayed in Figure 6. The smaller inner diameters significantly improve the peak pullout force and produce the profiles in Figure 6. As the inner diameter increases, the ISS decreases from Table 2. The ISS in case 4 is 1.17 times as large as that in case 6. It demonstrates the advantage of using CCNTs with small inner diameters due to their significantly higher ISS values. The reason is that the inner diameter of CCNTs influences *F_coil_*. When the inner diameter of CCNTs increases, the number of cycles of the CCNT decreases since *l_tot_* is determined. Since an extra force is needed to straighten the CCNT, less tensile force is required with fewer cycles. On the other hand, with larger inner diameters, the curvature decreases. Hence, the distance between the CCNT and the polymer matrix increases as displayed in Figure 5b. The interactions decrease, which results in a reduced tensile force. The trend is in good agreement with the corresponding results for wavy CNTs [27].

### 2.2. Effect of the Helix Angle

**Figure 7:**
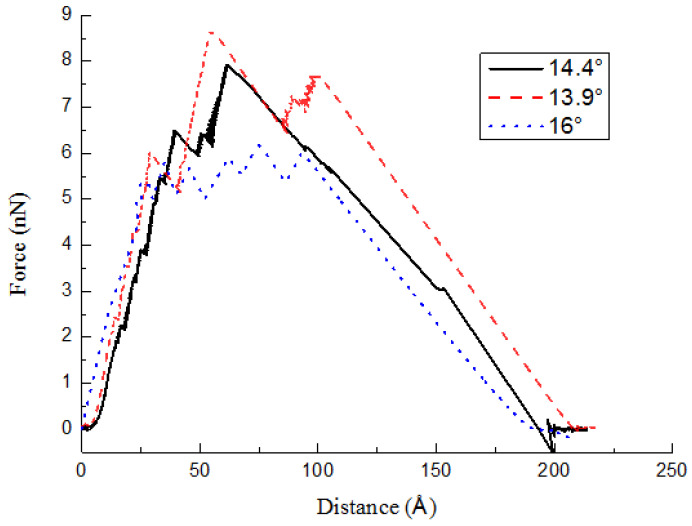
Pullout force–displacement curves for different helix angles.

Figure 7 shows that the maximal pullout force distinctly decreases with the increasing helix angles. Related parameters of CCNTs are illustrated in Table 3. The helix angle denotes the degree of helicity of the CCNT. It also controls the total length of CCNTs (*l_tot_*) embedded in the PE matrix with a fixed RVE height. Such results suggest that the greater helicity of the CCNT (i.e., smaller helix angles) can effectively enhance the maximal pullout force. Correspondingly, variations of the ISS are given in Table 3. The behavior of CCNTs is different from that in previous research with straight CNTs [16]. In previous research, the pullout profiles show very little variability, and the maximum pullout force is identical for different lengths. It can be inferred that the maximum pullout force remains relatively unchanged for CNTs with different lengths [16]. However, in the present research, the maximal tensile force of CCNTs increases with the reduced helix angle. The *F_coil_* is related to the total length of the CCNT embedded in the matrix. The CCNT/PE interface debonding force and the friction force increase with the CCNT length. With a greater *l_tot_*, more atoms in CCNTs interact with the PE matrix, which causes a larger interface. Hence, a greater tensile force is required to pull the CCNT out of the PE matrix. The trend is in good agreement with the corresponding results for wavy CNTs [27]. During the pullout process, the debonding of the CCNT/PE interface and the subsequent frictional sliding between CCNTs and the PE matrix consume energy. The CCNT with smaller helix angles consumes a larger amount of energy according to Figure 7, which means greater toughness when the CCNT with a smaller helix angle is adopted. The results in Table 3 indicate that the helix angle of the CCNT can also effectively influence the values of ISS. With the increased helix angles, the number of cycles of the CCNT decreases. A smaller force is required to straighten the CCNT. Hence, the ISS decreases. It also demonstrates the advantage of using CCNTs with small helix angles due to their significantly higher ISS values.

### 2.3. Effect of the Tube Diameter

**Figure 8:**
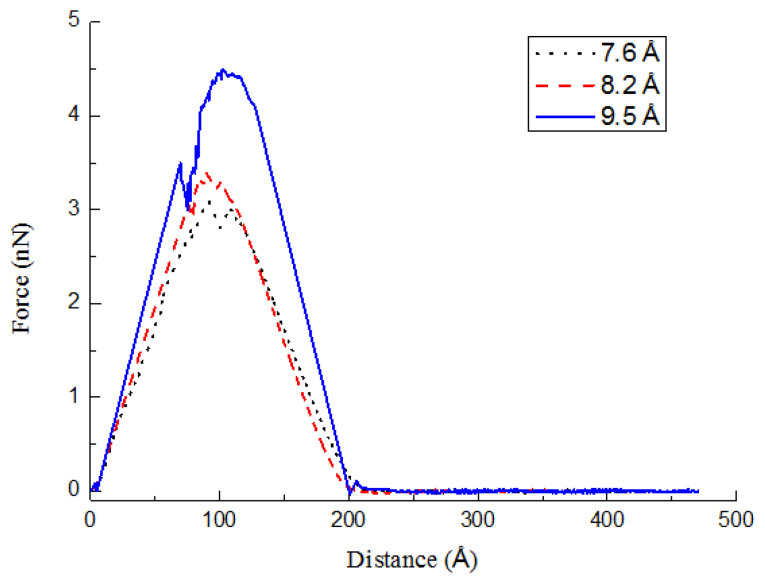
Pullout force–displacement curves for different diameters of CCNTs.

The size of the nanotube cross-section also distinctly influences the pullout behaviors of the CCNT. In Figure 8, the force–displacement curves with different tube diameters are plotted. Here, cases 9, 10, and 11 are investigated. Related geometric parameters of CCNTs are listed in Table 4. It can be found that the maximal tensile force of the pullout process goes up by increasing the cross-section size. In previous research, a greater diameter of straight CNTs increases the pull-out force [33]. The trend is in good agreement with the corresponding results for straight CNTs [33]. Meanwhile, the slope of the force–displacement curves rises with the tube diameters in Figure 8. The area below the force–displacement curves when *d_c_* = 0.95 nm is greater than that when *d_c_* = 0.82 nm or 0.76 nm, which suggests that a larger nanotube causes greater toughness.

**Figure 9:**
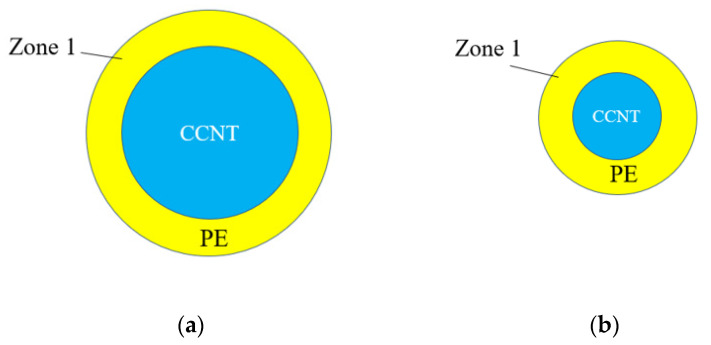

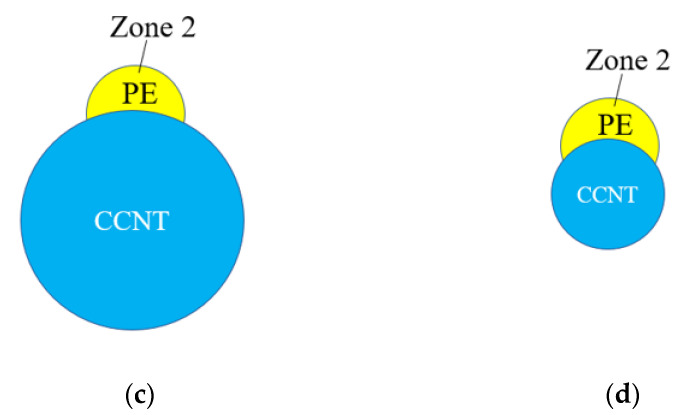
The CCNT and influenced PE matrix with different tube diameters. (**a**) Zone 1 of a large CCNT (**b**) Zone 1 of a small CCNT (**c**) Zone 2 of a large CCNT (**d**) Zone 2 of a small CCNT.

The reason for the increase in the maximal tensile force mainly lies in that the tube diameter increases the interaction zone between the CCNT and PE, as shown in Figure 9. The PE molecules that are influenced by CCNT are exhibited as Zone 1 in Figure 9. The influenced PE molecules are more with larger tube diameters (see Figure 9a) than that with smaller tube diameters (see Figure 9b). On the other hand, it is easier to straighten a smaller tube than a larger tube. However, the ISS decreases with the rising tube diameter. The ISS is related to the interaction between one atom in the CCNT and the influenced PE matrix. The influenced PE molecules by one C atom are displayed as Zone 2 in Figure 9. Since the influenced atoms by one C atom in the CCNT are more in Figure 9d than in Figure 9c, the ISS goes up when the tube diameter reduces. This research may also contribute to other high-aspect ratio sustainable biomaterials, which are complementary alternatives to carbon nanotubes.

## 3. Methods and Materials

Here, the large-scale atomic/molecular massively parallel simulator (LAMMPS) is adopted in the MD simulations [52]. The adaptive intermolecular reactive empirical bond-order potential (AIREBO) is utilized in the interatomic interactions of carbon nanotubes [53]. The parameters of carbon atoms come from previous research [54,55,56]. The polymeric matrix is described by the united-atom model using the Dreiding force field, whose functional form and parameters are listed in Table 5 [57]. The Lennard–Jones potential is utilized in the interatomic interaction between the PE and the CCNT. Table 5 shows the form and the parameters of the force field [54,58]. The cutoff is set at 10 Å [54].

### 3.1. Modeling the CCNT/PE Interface

**Figure 10:**
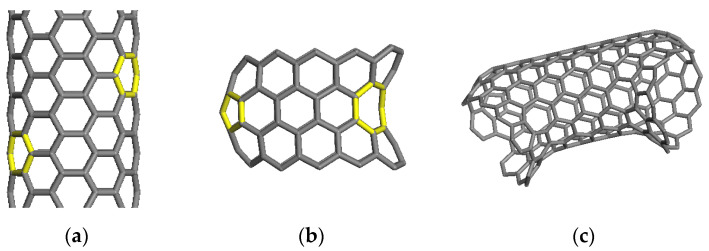
Procedures of constructing the CCNT model. The yellow rings are the introduced pentagons and heptagons. (**a**) Selecting the hexagonal rings (**b**) Introducing pentagons and heptagons (**c**) Final network.

In the beginning, atomistic models of CCNTs are constructed. One pair of pentagons and another pair of heptagons are first individually introduced in two sides of CNTs by adjusting the local topological structures of the two pairs of originally hexagonal rings as exhibited in Figure 10a. Pentagons form a cone defect, while heptagons result in a saddle defect, as is displayed in Figure 10b. Due to the strain energy induced by the pentagons and heptagons, the CNT becomes bent around the defect site after relaxation. A CCNT is formed based on the combination of basic structural segments. As illustrated in Figure 10c, two segments are connected to make the combined structure spiral. The structure in Figure 10c can be further used as a building block to construct complete CCNTs. By changing the tube length at the two ends of the basic segment or varying the nanotube diameter, different geometries of a CCNT are obtained. The CCNT exhibits a polygonal shape, which agrees with the experimental observation [59]. The periodic arrangement of pentagons and heptagons in the hexagonal network matches the features of the CCNT from previous research [60,61,62].

**Figure 11:**
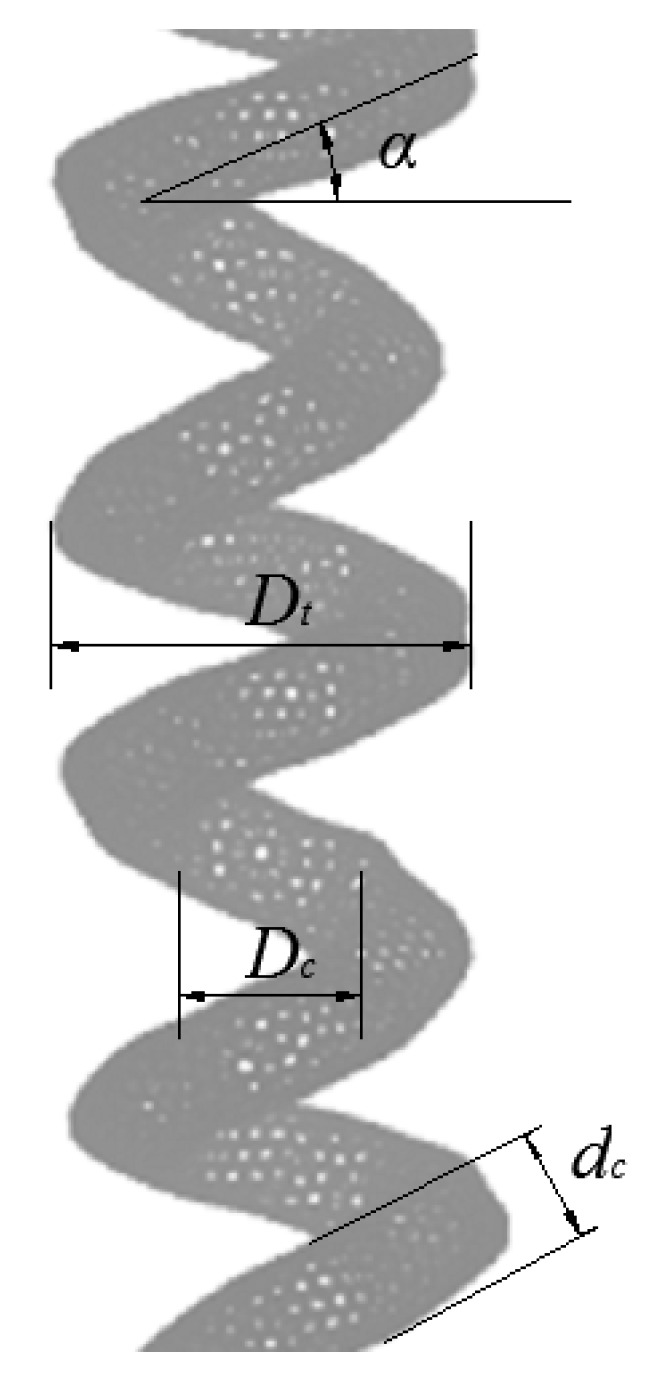
Geometrical parameters of a CCNT.

By using the method mentioned above, the CCNT model can be constructed as displayed in Figure 11. The related parameters are also presented in Figure 11. The parameters are chosen based on previous literature [7]. Here, the inner diameter (*D_c_*), the helix angle (*α*) and the tube diameter (*d_c_*) of CCNTs are investigated. The inner diameter (*D_c_*) means the diameter of the inner cylinder, which is surrounded by the carbon nanocoil, and can be obtained by *D_c_* = *D_t_ −* 2*dc*. *D_t_* means the outer diameter of the CCNT.

**Figure 12:**
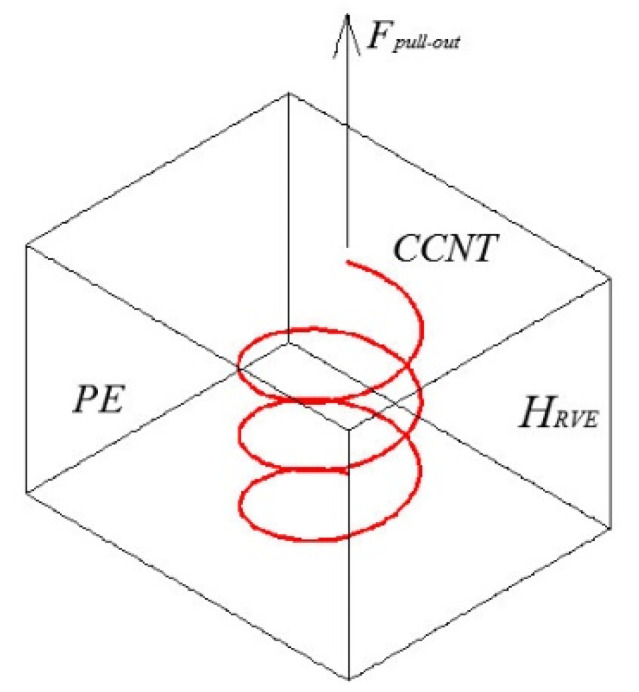
The RVE and the CCNT. The CCNT is shown in red.

Then, a series of CNTs (i.e., cases 1 and 2) and CCNTs (i.e., cases 3–11) are built, which are illustrated in Table 6. Case 1 is modeled for comparison with previous research [33], which has a diameter of 8.14 Å. Two MD simulations of the pullout test of the straight CNT (i.e., case 2) and the CCNT (i.e., case 3) are conducted to determine quantitatively the effect of shape on the pullout force and ISS. Cases 4–6 are modeled to study the effect of the inner diameters of the CCNT. Cases 4–6 are modeled to investigate the effect of the helix angle. Cases 9–11 are modeled to explore the effect of the tube diameter. For the CCNT, the total length can be expressed as *l_tot_* = *H_RVE_*/sin(*α*), where *H_RVE_* denotes the height of a representative volume element (RVE). After developing the CCNT model, the CCNT/PE interface model is built. Each RVE has one CCNT and 290 PE chains. Each PE chain contains 150 carbon atoms. The CCNT is set in the middle of the RVE as exhibited in Figure 12. The size of the RVE in each case is adjusted by forming the height as 5 nm. The total length of the CCNT after being straightened is about 20 nm, which is used in the pullout test with straight CNTs [44]. The width and length of the RVE are selected to be large enough so that the obtained results are independent of the size of the RVE. In the MD simulations, the periodic boundary condition is applied in 𝑥-𝑦 plane and the free boundary condition is assigned to 𝑧 direction. In the z direction, a very large blank space is set to make room for the pulled-out CCNT.

### 3.2. MD Simulation Procedures

MD simulations of the pullout tests of CCNTs from the PE matrix are conducted by steered molecular dynamics [63]. A moving spring force is applied to the atoms at the head end of CCNTs [64] with the elastic spring constant *K*_spring_ = 100 eV/Å^2^ [46]. The tensile force of the pullout test can be recorded, as well as the displacement. The complete procedure for conducting the pullout simulations is illustrated as follows.

After modeling the CCNT and the polymetric matrix individually, the initial MD models of the PE and CCNTs are equilibrated by the conjugate gradient method [65]. The conjugate gradient method is one of the most popular and well-known iterative techniques for solving systems of sparsely symmetric positive definite linear equations. It is an important optimization algorithm and is widely adopted for structural optimization in MD. The energy-based stopping tolerance is set as etol = 10^−30^ and the force-based stopping tolerance is set as ftol = 10^−30^ eV/°A. More details can be found in previous research [66]. Then, the PE model and the CCNT model are put together. A specific number of the PE structures are dispersed randomly around a CCNT to build the nanocomposite using the Packmol package. Another PE model without the CCNT is also prepared as the control sample. The PE model without CCNTs is built just for the validation of the method with experimental results. After equilibrium, the density of the PE control sample without the CCNT is 0.83 g/cm^3^, which is close to the data reported previously (c.f. Figure 3 in [67]). The calculated glass transition temperature (Tg) value is 252 K, which is in the experimental temperature range of 190–300 K in other research work [68,69]. The simulation domain is gradually compressed to the targeted size. At the beginning of each compression stage, the coordinates of all atoms are remapped to fit inside the updated domain. Then, the configuration is relaxed to reach equilibrium with the minimum potential energy. Geometry optimization of materials at the micro level is a prerequisite in most studies using molecular dynamics. The potential energy is a function of the coordinates of atoms. By changing the coordinate, the potential energy of the material can be minimized. This leads us to define the minimization problem. In the case of periodic systems, the geometry optimization is typically done using Cartesian coordinates. The number of degrees of freedom required for optimization is just 3*N* − 3, where *N* is the number of atoms in a single unit cell. The calculation is complete when the iterations have converged. They will be sufficiently close to their minimum structures, so that further full optimization only takes very few steps and the energetics will not change substantially. The complete description can be found in previous research [70]. The obtained system is further equilibrated at 300 K using the constant temperature and volume canonical (NVT) ensemble over 200 ps with 1 fs time step. Next, the equilibration is continued for another 200 ps by the isothermal-isobaric (NPT) ensemble at 300 K and 1 atm. The obtained system with the CCNT has a density of 1.0–1.1 g/cm^3^, which is in accordance with previous research [71]. In this study, an agreement between the calculated physical properties of the MD models, i.e., density and Tg value, and the experimental results indicates that the stable and equilibrated MD models are achieved. Three models with different initial configurations are built to obtain the average ISS of each case.

Finally, atoms at the head end of the CCNT are pulled out from the PE matrix at a constant velocity of 1 × 10^−4^ Å/fs while the nanotube is equilibrated in the NVT ensemble at 300 K [72]. The configuration of the tensile simulation is displayed in Figure 12. The atoms of the polymer matrix are fixed during the pullout simulation [33]. The ISS is adopted as the representative of the interfacial properties between CCNTs and the polymer, which is calculated by [45,73]:

(2) $$\mathrm{ISS}=\frac{F_{pull-out}}{A_{interface}}=\frac{F_{pull-out}}{\pi dl_{tot}}$$

where, *F_pull_*_-*out*_ is the maximum pullout force, *A_interface_* is the area of interfaces, *d* is the diameter of tubes and *l_tot_* is the embedded CCNT length.

## 4. Conclusions

The pullout test of CCNTs has been simulated using MD to investigate the properties of the CCNT/PE interface. Different geometries of CCNTs, like the inner diameters, helix angles, and tube diameters, are considered. The results reveal that the maximal tensile force in the pullout tests is reduced by increasing CCNT inner diameters, increasing the helix angles and decreasing nanotube diameters. The distance between CCNTs and the polymer matrix is varied, and the less interfacial distance favors the greater ISS. Decreasing the inner diameter of the CCNT, the helix angle, and the tube diameter increases the ISS. The related enhancement mechanism has been illustrated. The present study helps to understand the interfacial adhesion behavior between polymers and CCNTs and is expected to contribute to the development of CCNT reinforced polymer composites. This research may also contribute to other high-aspect ratio sustainable biomaterials, which are complementary alternatives to carbon nanotubes. For example, fiber-reinforced composites (FRCs) [74] or cellulose nanocrystals (CNCs) [75] meet the criteria and can have potential applications in the preparation of composites for dentistry [76] and food packaging [77] fields, respectively.

In order to better understand the intermolecular interactions between neighboring CCNTs that eventually could influence the interfacial adhesion properties, the interaction between CCNTs should be considered in the pulling-out event. The dynamic simulation studies considering several CCNTs in the same pulling out event will be investigated in our following research.

## Figures and Tables

**Table 1:** Geometries and pullout results for the straight CNT and the CCNT.

Case #	Type	Outer Diameter (*D_t_*) (Å)	Helix Angle (*α*) (°)	Tube Diameter (*d_c_*) (Å)	Inner Diameter (*D_c_*) (Å)	Maximal Pullout Force (nN)	ISS (MPa)
2	CNT	11.3	-	11.3	-	1.946	127
3	CCNT	37.4	14.4	11.3	14.8	7.917	149

**Table 2:** Pullout force and ISS for different inner diameters.

Case #	Outer Diameter (*D_t_*) (Å)	Helix Angle (*α*) (°)	Tube Diameter (*d_c_*) (Å)	Inner Diameter (*D_c_*) (Å)	Maximal Pullout Force (nN)	ISS (MPa)
4	32.6	16	11.3	10	7.35	155
5	36.6	16	11.3	14	6.61	142.1
6	37.4	16	11.3	14.8	6.167	132.5

**Table 3:** Pullout force and ISS for different helix angles.

Case #	Outer Diameter (*D_t_*) (Å)	Helix Angle (*α*) (°)	Tube Diameter (*d_c_*) (Å)	Inner Diameter (*D_c_*) (Å)	Maximal Pullout Force (nN)	ISS (MPa)
6	37.4	16	11.3	14.8	6.167	132.5
7	37.4	14.4	11.3	14.8	7.917	149
8	37.4	13.9	11.3	14.8	8.631	153.8

**Table 4:** Pullout force and ISS for different tube diameters.

Case #	Outer Diameter (*D_t_*) (Å)	Helix Angle (*α*) (°)	Tube Diameter (*d_c_*) (Å)	Inner Diameter (*D_c_*) (Å)	Maximal Pullout Force (nN)	ISS (MPa)
9	29.4	14	7.6	14.2	3.12	168
10	30	14	8.2	13.6	3.32	163
11	29.5	14	9.5	10.5	4.45	160

**Table 5:** Function form of force field and potential parameters used for PE.

Interaction	Form	Parameters
Bond	$E=\frac{1}{2}k_{b}{\left(l-l_{eq}\right)}^{2}$	$k_{b}=2000$ kJ/mol Å, *l_eq_* = 1.53 Å
Angle	$E=\frac{1}{2}k_{\theta }{\left(cos\left(\theta \right)-cos\left(\theta_{eq}\right)\right)}^{2}$	$k_{\theta }$ = $510\;\mathrm{kJ}/\mathrm{mol},\;\theta_{eq}$ = 110°
Torsional	$E=\frac{1}{2}\sum_{n=0}^{3}k_{n}cos^{n}\varphi$	*k*_0_ = 14.477, *k*_1_ = 37.594, *k*_2_ = 6.493, *k*_3_ = 58.499 (kJ/mol)
Non-bonded	$u=4\varepsilon \left\{{\left(\frac{\sigma }{r}\right)}^{12}-{\left(\frac{\sigma }{r}\right)}^{6}\right\}$	*ε* = 0.468 kJ/mol, σ = 4.01 Å, *r_c_* = 10.0 Å

**Table 6:** Parameters of CNTs and CCNTs.

Case #	Type	Outer Diameter (*D_t_*) (Å)	Helix Angle (*α*) (°)	Tube Diameter (*d_c_*) (Å)	Inner Diameter (*D_c_*) (Å)
1	CNT	8.14	-	8.14	-
2	CNT	11.3	-	11.3	-
3	CCNT	37.4	14.4	11.3	14.8
4	CCNT	32.6	16	11.3	10
5	CCNT	36.6	16	11.3	14
6	CCNT	37.4	16	11.3	14.8
7	CCNT	37.4	14.4	11.3	14.8
8	CCNT	37.4	13.9	11.3	14.8
9	CCNT	29.4	14	7.6	14.2
10	CCNT	30	14	8.2	13.6
11	CCNT	29.5	14	9.5	10.5

## Data Availability

Data is contained within the article.
